# Supplementary material for: Structural and mechanistic diversity in p53-mediated regulation of organismal longevity across taxonomical orders
Source: bioRxiv. 2024 Aug 6:2024.08.05.606567. Preprint. [Version 1] doi: 10.1101/2024.08.05.606567 (PMC11326148; doi:10.1101/2024.08.05.606567)
Supplement: Supplement 1 [file NIHPP2024.08.05.606567v1-supplement-1.pdf]

bioRxiv preprint doi: https://doi.org/10.1101/2024.08.05.606567; this version posted August 6, 2024. The copyright holder for this preprint (which was not certified by peer review) is the author/funder, who has granted bioRxiv a license to display the preprint in perpetuity. It is made available under aCC-BY 4.0 International license.

Supporting Information

Organism			Protein sequence accession numbers									
Scientific name	Common name	Average lifespan (years)	p53	Smad2	Smad3	Rbl2	Npm1	Rpl11	MDM2	Pras40	Akt	Klf4
*Callithrix jacchus**	Common marmoset	10	A0A2R8MYD9	A0A2R8M5L4	F7DCC1	U3DXJ5	F7FL37	U3EPN5	F6UPT1	F6RUS2	U3C443	F7IJX6
*Carlito syrichta**	Philippine tarsier	13	A0A1U7U5H4	/	/	/	/	A0A1U7TUA5	A0A3Q0DPE7	/	/	A0A1U7TCZ4
*Microcebus murinus**	Gray mouse lemur	14	A0A8B7H7E6	A0A8B7F143	A0A8C5YBL9	A0A8B7GYT6	A0A8C5YAS8	A0A8C5XZX6	A0A8C5V8R7	A0A8C6EKZ4	A0A8C5VV71	A0A8B7EFL3
*Mandrillus leucophaeus**	Drill	28	A0A2K6AAH4	A0A2K5ZHC1	A0A2K5YMS3	A0A2K5ZL30	A0A2K5YDB8	A0A2K5YMD0	A0A2K6A872	A0A2K6A7P6	A0A2K5ZJA3	/
*Macaca fuscata fuscata**	Japanese macaque	30	P61260	/	/	/	/	/	/	/	/	/
*Sapajus apella**	Tufted capuchin	40	A0A6J3I233	A0A6J3GBM2	A0A6J3F4R4	A0A6J3G130	A0A6J3GDU9	A0A6J3JQA4	A0A6J3IMI1	A0A6J3GQB8	A0A6J3GYY1	A0A6J3EU19
Gorilla gorilla gorilla	Gorilla	47	G3R2U9	G3QPV6	A0A2I2ZDG8	G3RZ75	A0A2I2YQY6	G3RWD2	A0A2I2ZWM5	G3S586	G3RB32	G3SJ37
*Pan troglodytes**	Chimpanzee	56	A0A2I3S1V8	H2QEH9	A0A2I3TN10	K7C590	A0A2J8LXX4	A0A2I3TKT2	H2R792	K7AMC0	K7AGW5	H2QXN2
Homo sapiens *	Human	76	P04637	Q15796	P84022	Q08999	P06748	P62913	Q00987	Q96B36	P31749	O43474
*Macaca mulatta**	Indochinese rhesus macaque	26	P56424	F7CP27	A0A1D5Q1C9	A0A1D5QDE7	F7AWJ2	F6PJ30	G7N7L5	A0A5F8AIB7	G7MWI7	A0A5F8AGZ8
*Macaca fascicularis**	Crab-eating macaque	38	P56423	/	/	/	/	A0A2K5UHI1	A0A2K5W2P1	/	/	A0A2K5VB57
*Pan paniscus**	Bonobo	41.5	A0A2R9A5P4	/	/	/	/	A0A2R9AUR7	A0A2R9AQ86	A0A2R9CMW9	A0A2R9BZ66	A0A2R8ZF10
Saimiri boliviensis boliviensis	Black-capped squirrel monkey	17.5	A0A2K6SLC5	A0A2K6V827	A0A2K6SKP9	A0A2K6V4D2	A0A2K6UT13	A0A2K6V210	A0A2K6S797	A0A2K6UND0	A0A2K6SGS8	A0A2K6SJ96

bioRxiv preprint doi: https://doi.org/10.1101/2024.08.05.606567; this version posted August 6, 2024. The copyright holder for this preprint (which was not certified by peer review) is the author/funder, who has granted bioRxiv a license to display the preprint in perpetuity. It is made available under aCC-BY 4.0 International license.

Theropithecus gelada	Gelada	20.8	A0A8D2F964	A0A8D2GAI2	A0A8D2G920	A0A8D2FRL2	A0A8D2K0P5	A0A8D2EEE2	A0A8D2K6W8	A0A8D2E2Q4	A0A8D2F2J1	A0A8D2E5U5
*Rhinopithecus bieti **	Black-and-white snub-nosed monkey	23	A0A2K6LYM5	A0A2K6KLL7	A0A2K6K5G6	A0A2K6JU68	A0A2K6MQI9	A0A2K6K899	A0A2K6KDN6	A0A2K6KJD1	A0A2K6KHG5	A0A2K6JXF0
Papio anubis	Olive baboon	25.2	A0A096P2R8	A0A096NP77	A0A096NMF6	A0A096NIB6	A0A2I3MVB3	UPI000440407B	A0A2I3NF10	A0A0A0MU05	A0A2I3N0A6	A0A8I5R2U0
*Cebus imitator**	Panamanian white-faced capuchin	45	A0A2K5RMZ8	/	/	/	/	/	/	/	/	/
*Pongo abelii**	Sumatran orangutan	55	A0A2J8RWD8	/	/	/	/	/	/	/	/	/
*Chlorocebus sabaeus**	Green monkey	12	A0A0D9RG12	/	/	/	/	/	/	/	/	/

S1 Table. Primates included in the study.

Organisms in order Primates included in the RES, SIFT, and/or PEPPI studies, their average lifespans, and UniPro or NCBI GenBank's protein accession numbers. * indicates those organisms whose p53 sequences are used in alignment for RES study.

Organism			Sequence accession numbers									
Scientific name	Common name	Average lifespan (years)	p53	Smad2	Smad3	Rbl2	Npm1	Rpl11	MDM2	Pras40	Akt	Klf4
*Thunnus albacares**	Yellowfin tuna	9	XP_044196743.1	UPI001C4B2943	UPI00054C1478	UPI001CF64C10	/	UPI001C4D5AE3	UPI001CF6DDC0	/	/	UPI001CF6E266
*Perca flavescens**	Yellow perch	12	A0A484CCM0	UPI00106EAF7A	A0A484DP09	A0A484D3R4	A0A484CM55	A0A484CRN0	A0A484BZ23	A0A6A5ENB6	A0A484C4T4	A0A484CJE2

bioRxiv preprint doi: https://doi.org/10.1101/2024.08.05.606567; this version posted August 6, 2024. The copyright holder for this preprint (which was not certified by peer review) is the author/funder, who has granted bioRxiv a license to display the preprint in perpetuity. It is made available under aCC-BY 4.0 International license.

Dicentrarchus labrax	European seabass	15	A0A8C4EKX1	A0A8C4E8G4	A0A8C4F4T0	A0A8P4KC99	A0A8C4I739	UPI0021F50FAC	A0A8C4DZ38	A0A8C4DEU5	A0A8C4DG29	A0A8C4HU78
*Platichthys flesus**	European flounder	15	O12946	UPI002DB75018	/	/	/	UPI0011C10C13	UPI002DB897B8	/	/	UPI002DBCCB49
*Sander lucioperca**	Zander	16	A0A8C9ZEJ7	A0A8D0D734	A0A8C9ZTJ9	A0A8D0CS09	A0A8C9XKY4	A0A8D0AAC8	A0A8C9XZT3	A0A8C9YHW4	A0A8C9XJN6	A0A8C9XNT1
*Scomber scombrus**	Atlantic mackere	17	UPI002DD90AFB	UPI002DD915F3	/	UPI002DD91C6B	/	UPI00016333A1	UPI002FEBFDE1	/		UPI002DD90CB4
*Perca fluviatilis**	European perch	22	A0A6A5F0Y3	A0A6A5EE76	A0A6A5FEG0	A0A6A5FA52	A0A6A5ESY6	A0A6A5ELS6	A0A6A5DZX5	/	A0A6A5DQU5	A0A6A5EH34
*Epinephelus coioides**	Orange-spotted groupe	22	F8RKR1	/	/	/	A0A1D8V787	/	/	/	/	/
*Labrus bergylta**	Ballan wrasse	29	A0A3Q3GRI3	UPI000F31D7C5	A0A3Q3MUV6	A0A3Q3LLZ5	A0A3Q3LSC0	UPI000F30F9F7	A0A3Q3FQT2	A0A3Q3E1S6	A0A3Q3GN09	A0A3Q3G1D1
*Anabas testudineus**	Climbing perch	7	A0A3Q1JCZ8	A0A7N6BK16	A0A3Q1J1S9	A0A3Q1IKN7	A0A7N6AAM7	A0A3Q1IK23	A0A3Q1JLB4	A0A3Q1JP30	UPI00194B08E9	A0A3Q1J2Y9
Amphiprion percula	Orange clownfish	18	A0A3P8T0X9	A0A3P8RXC7	UPI000F387D83	/	A0A3P8SIS1	A0A3P8TH83	A0A3P8TU83	A0A3P8TXZ2	A0A3P8RL25	A0A3P8TF24

bioRxiv preprint doi: https://doi.org/10.1101/2024.08.05.606567; this version posted August 6, 2024. The copyright holder for this preprint (which was not certified by peer review) is the author/funder, who has granted bioRxiv a license to display the preprint in perpetuity. It is made available under aCC-BY 4.0 International license.

*Sparus aurata**	Gilt-head bream	11	A0A671VUB9	A0A671XQA2	A0A671TKP9	A0A671TJ72	A0A671TUQ4	A0A671YTZ8	A0A671Y7M9	A0A671XJ86	A0A671XJ74	A0A671VEU0
*Acanthochromis polyacanthus**	Spiny chromis	6	A0A3Q1HGV2	/	/	/	/	/	/	/	/	/
*Parambassis ranga**	Indian Glass Fish	5.5	A0A6P7K8V8	/	/	/	/	/	/	/	/	/
*Sphaeramia orbicularis**	Orbiculate cardinalfish	4	A0A673CZK1	/	/	/	/	/	/	/	/	/
*Salarias fasciatus**	Lawnmower blenny	3	A0A672G194	/	/	/	/	/	/	/	/	/
*Thunnus maccoyii**	Southern bluefin tuna	20	UniRef100_UPI001C4B3AFC	/	/	/	/	/	/	/	/	/

S2 Table. Perciformes included in the study.

Organisms in order Perciformes included in the RES, SIFT, and/or PEPPI studies, their average lifespans, and UniPro or NCBI GenBank's protein accession numbers. * indicates those organisms whose p53 sequences are used in alignment for RES study.

Organism			Sequence accession numbers
Scientific name	Common name	Average lifespan (years)	p53
Mus musculus	House mouse	2	P02340

bioRxiv preprint doi: https://doi.org/10.1101/2024.08.05.606567; this version posted August 6, 2024. The copyright holder for this preprint (which was not certified by peer review) is the author/funder, who has granted bioRxiv a license to display the preprint in perpetuity. It is made available under aCC-BY 4.0 International license.

Cricetulus griseus	Chinese hamster	2.5	O09185
Mesocricetus auratus	Golden hamster	2.5	Q00366
Cavia porcellus	Guinea pig	5	Q9WUR6
Octodon degus	Common degu	6.5	A0A6P3F490
Chinchilla lanigera	Long-tailed chinchilla	10	A0A8C2YMX2
Castor canadensis	American beaver	11	A0A250YHC8
Spalax judaei	Middle East blind mole-rat	21	Q68VB0
Heterocephalus glaber	Naked mole-rat	31	G5B5D6
Mastomys natalensis	Natal multimammate mouse	0.25	P89002
Rattus norvegicus	Brown rat	3.8	P10361
Peromyscus maniculatus	Deer Mouse	8.7	A0A8C8TJT9
Meriones unguiculatus	Mongolian gerbil	6.3	Q920Y0
Ictidomys tridecemlineatus	Thirteen-lined ground squirrel	7.9	I3N5N2
Marmota monax	Groundhog	14	O36006

S3 Table. Rodents included in the study.

Organisms in order Rodentia included in the RES and SIFT studies, their average lifespans, and UniPro or NCBI GenBank's protein accession numbers.

Organism			Sequence accession numbers
Scientific name	Common name	Average lifespan (years)	p53

bioRxiv preprint doi: https://doi.org/10.1101/2024.08.05.606567; this version posted August 6, 2024. The copyright holder for this preprint (which was not certified by peer review) is the author/funder, who has granted bioRxiv a license to display the preprint in perpetuity. It is made available under aCC-BY 4.0 International license.

Neovison vison	American mink	11.4	A0A8C7A9R2
Vulpes vulpes	Red fox	12	A0A3Q7TIN0
Canis lupus dingo	Dingo	14	A0A8C0K1L6
Nyctereutes procyonoides	Common raccoon dog	15.6	A0A811ZM26
Canis lupus familiaris	Dog	20.6	Q29537
Neomonachus schauinslandi	Hawaiian monk seal	25	A0A2Y9HEV7
Callorhinus ursinus	Northern fur seal	25	A0A3Q7NG00
Panthera leo	Lion	28	A0A8C8Y413
Felis catus	Cat	30	P41685
Ursus maritimus	Polar bear	38.2	A0A384BVC2
Zalophus californianus	California sea lion	30	A0A6J2FNY3
Lynx rufus	Bobcat	32.3	UPI001F1287F1
Enhydra lutris	Sea otter	19	A0A2Y9L4Q2
Panthera pardus	Leopard	22	A0A9V1DWE6
Suricata suricatta	Meerkat	12.5	A0A673UVH2

S4 Table. Carnivores included in the study.

Organisms in order Carnivora included in the RES and SIFT studies, their average lifespans, and UniPro or NCBI GenBank's protein accession numbers.

Organism			Sequence accession number
Scientific name	Common name	Average lifespan (years)	p53
Lipotes vexillifer	Baiji	24	A0A340X8E6
Delphinapterus leucas	Beluga whale	40	Q8SPZ3
Tursiops truncatus	Common bottlenose dolphin	45	A0A2U4C2U9
Monodon monoceros	Narwhal	50	A0A4V5P9N3

bioRxiv preprint doi: https://doi.org/10.1101/2024.08.05.606567; this version posted August 6, 2024. The copyright holder for this preprint (which was not certified by peer review) is the author/funder, who has granted bioRxiv a license to display the preprint in perpetuity. It is made available under aCC-BY 4.0 International license.

Globicephala melas	Long-finned pilot whale	60	UPI00293D9E1D
Eubalaena glacialis	North Atlantic right whale	67	UPI002A5A2F50
Physeter macrocephalus	Sperm whale	68	A0A455C1G1
Balaenoptera musculus	Blue whale	85	A0A8C0DFA9
Balaenoptera physalus	Fin whale	114	A0A6A1Q3Q7
Balaenoptera acutorostrata	Common minke whale	57	A0A383YUA7
Lagenorhynchus albirostris	White-beaked dolphin	40	XP_059988824.1
Mesoplodon densirostris	Blainville's beaked whale	27	XP_059937197.1
Delphinus delphis	Short-beaked common dolphin	25	XP_059854544.1
Kogia breviceps	Pygmy sperm whale	17	XP_058904949.1
Phocoena phocoena	Harbour porpoise	13	XP_065753055.1

S5 Table. Cetaceans included in the study.

Organisms in order Catacean included in the RES and SIFT studies, their average lifespans, and UniPro or NCBI GenBank's protein accession numbers.

Organism			Sequence accession number
Scientific name	Common name	Average lifespan (years)	p53
Ovis aries	Sheep	11	P51664
Sus scrofa	Wild boar	12	Q9TUB2
Muntiacus muntjak	Southern red muntjac	17	A0A5N3WLG9

bioRxiv preprint doi: https://doi.org/10.1101/2024.08.05.606567; this version posted August 6, 2024. The copyright holder for this preprint (which was not certified by peer review) is the author/funder, who has granted bioRxiv a license to display the preprint in perpetuity. It is made available under aCC-BY 4.0 International license.

Bos taurus	Cattle	20	P67939
Capra hircus	Goat	20.8	A0A452G0A3
Odocoileus virginianus texanus	Texas white-tailed deer	23	A0A6J0VGP1
Vicugna pacos	Alpaca	25	A0A6I9IU1
Bison bison bison	Plains bison	33.5	A0A6P3HQA5
Camelus bactrianus	Bactrian camel	35	A0A9W3H045
Hippopotamus amphibius kiboko	Hippopotamus	61	XP_057572051.1
Camelus dromedarius	Dromedary	45	A0A5N4D2L6
Bubalus bubalis	Domestic water buffalo	34.9	F6MDM8
Bos mutus grunniens	Wild yak	26.3	A0A0N7FDT7
Neophocaena asiaeorientalis asiaeorientalis	Yangtze finless porpoise	21.5	A0A341BQX3
Bos indicus	Zebu	20	P67938

S6 Table. Artiodactyles included in the study.
Organisms in order Artiodactyl included in the RES and SIFT studies, their average lifespans, and UniPro or NCBI GenBank’s protein accession numbers.

RES GitHub repository can be accessed at https://github.com/Romani20/RES. It includes the RES program, installation instructions, and fasta files to score p53 sequences involved in study.
